# Cyclopædia of Practice of Medicine

**Published:** 1876-03

**Authors:** 


CYCLOPÆDIA OF THE PRACTICE OF MEDICINE. Edited by Dr. H. Von Zeimssen, Professor of Clinical Medicine in Munich, Bavaria. Albert H. Rusk, M.D., New York, Editor American Edition. Wm. Wood & Co., 27 Great Jones Street, New York.

The fifth volume of this valuable work has been received. Upon the reception of former volumes we have said so much in praise of this great book that anything more in this direction would prove stale to our readers. We will add, however, that the fact is well established that no publication within the past half century has been received by the profession with such universal commendation as has the Cyclopædia of the Practice of Medicine. The fifth volume embraces the diseases of the respiratory organs, and is exhaustible.

The chapters on croupus pneumonia, catarrhal pneumonia and embolic pneumonia are by Prof. Jurgensen, of Tubingen. Those on hyperæmia, anæmia hæmorrhages, atelectasis, collapse, hypertropby, gangrene, new formations, and parisites of the lungs, are prepared by Prof. Hertz, of Amsterdam. Those on pulmonary consumption and acute milliary tuberculosis by Prof. Ruehle, of Boun; while Prof. Rindfleish has prepared the text on chronic and acute tuberculosis.

